# A Novel Rapid MALDI-TOF-MS-Based Method for Measuring Urinary Globotriaosylceramide in Fabry Patients

**DOI:** 10.1007/s13361-015-1318-4

**Published:** 2016-01-21

**Authors:** Fahad J. Alharbi, Tarekegn Geberhiwot, Derralynn A. Hughes, Douglas G. Ward

**Affiliations:** Institute of Cancer and Genomic Sciences, College of Medical and Dental Sciences, University of Birmingham, Birmingham, B15 2TT England; Queen Elizabeth Medical Centre, University Hospitals Birmingham NHS Foundation Trust, Birmingham, B15 2TH England; Department of Haematology, Lysosomal Storage Disorders Unit, Royal Free Hospital, London, NW3 2QG England

**Keywords:** MALDI-TOF-MS, Fabry disease, Biomarker, Gb3

## Abstract

Fabry disease is an X-linked lysosomal storage disorder caused by deficiency of α-galactosidase A, resulting in the accumulation of glycosphingolipids in various organs. Globotriaosylceramide (Gb3) and its isoforms and analogues have been identified and quantified as biomarkers of disease severity and treatment efficacy. The current study aimed to establish rapid methods for urinary Gb3 extraction and quantitation. Urine samples from 15 Fabry patients and 21 healthy control subjects were processed to extract Gb3 by mixing equal volumes of urine, methanol containing an internal standard, and chloroform followed by sonication and centrifugation. Thereafter, the lower phase was analyzed by MALDI-TOF MS and the relative peak areas of the internal standard and four major species of Gb3 determined. The results showed high reproducibility with intra- and inter-assay coefficients variation of 9.9% and 13.7%, respectively. The limit of detection was 0.15 ng/μL and the limit of quantitation was 0.30 ng/μL. Total urinary Gb3 levels in both genders of classic Fabry patients were significantly higher than in healthy controls (*p* < 0.0001). Gb3 levels in Fabry males were higher than in Fabry females (*p* = 0.08). We have established a novel assay for urinary total Gb3 that takes less than 15 min from start to finish.

**Electronic supplementary material:**

The online version of this article (doi:10.1007/s13361-015-1318-4) contains supplementary material, which is available to authorized users.

**Graphical Abstract:**
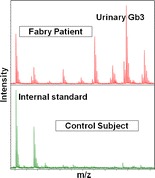
ᅟ

## Introduction

Fabry disease (FD) is a rare multi-systemic X-linked lysosomal storage disorder. This inborn error of glycosphingolipid metabolism results from the absence or deficiency of alpha-galactosidase A (α-GAL A, OMIM 301500), which is responsible for hydrolytic cleavage between the galactose residues in globotriaosylceramide (Gb3). The un-metabolized glycosphingolipids accumulate in various organs, resulting in a range of pathologies in the kidneys, heart, brain, eye, peripheral nervous system, and skin. Male patients show symptoms at an early age whereas females display symptoms when older. The precise incidence of FD is not known: the reported figures vary widely. The rarity of the disease and wide spectrum of symptoms displayed by Fabry patients make diagnosis based on clinical manifestation problematic. FD is currently diagnosed by measuring the activity of α-GAL A and/or by mutation analysis of the α-GAL A gene where enzyme analysis may not be informative in females [1–7].

Enzyme replacement therapy (ERT), using recombinant α-GAL A has become the standard treatment for symptomatic Fabry patients since 2001 [8–10]. However, the clinical benefit of this expensive treatment is limited, depending on the clinical stage at which therapy is initiated. Gb3 levels in plasma or urine can be used to monitor the efficacy of ERT and as a diagnostic biomarker although with limited sensitivity [11–17]. Gb3 levels in biological samples decrease considerably within 2 wk of starting ERT but later increase in some patients probably due to development of α-GAL A antibodies and do not show a good correlation with effects on clinical symptoms or organ function [11, 12].

Gb3 and its isoforms/analogues have been detected at elevated levels in plasma and urine of Fabry patients using mass spectrometric approaches [14, 15, 18–21]. Several analytical methods have been reported for urinary Gb3 extraction and analysis. Auray-Blais and co-workers originally used filter paper discs saturated with urine, which were dried and Gb3 extracted with methanol and analyzed by LC-MS/MS [14]. Later, Krüger and colleagues [15] combined liquid extraction/protein precipitation and solid phase extraction for urinary Gb3 extraction before LC-MS/MS analysis. Despite being fairly complex and time-consuming, these methods generated invaluable data on urinary Gb3 levels in FD. Recently, an advanced multiplex method has been established by Auray-Blais team where they were successful in measuring the levels of different Gb3 isoforms and analogues using liquid–liquid extraction and LC-MS/MS [19, 20].

In this study, we report the development of a rapid, robust assay for Gb3 based on a novel liquid–liquid extraction followed by MALDI-TOF MS, and validate it using urine from patients with classic FD and healthy control subjects.

## Materials and Methods

### Patient Samples

Fabry patients with diagnoses confirmed by mutation analysis and enzyme activity test were enrolled in the study after giving written informed consent (ethical approval reference: 09/H1010/75). Urine samples were collected from 15 classic Fabry patients and 21 age- and gender-matched healthy controls (patient details are shown in Supplemental Information Table S1). Urine samples were stored at –80 °C until further processing without any centrifugation or filtration. Classic Fabry patients are defined as having typical Fabry disease manifestations and known classic mutations with the deficiency of α-GAL A enzyme activity (<1%).

### Chemicals

HPLC grade water and acetonitrile (ACN) were purchased from (VWR International-UK). Acetone was purchased from (Fisher Scientific, Loughborough, UK). HPLC grade methanol (MeOH, ≥99.9%), HPLC grade chloroform (CHCl_3_, ≥99.9%), 5-chloro-2-mercaptobenzothiazole (5C2M), 2-mercaptobenzothiazole, 6-aza-2-thiothymine, 2-(4-hydroxyphenylazo) benzoic acid, 2,5-dihydroxy benzoic acid (DHB), super-DHB, sinapic acid, 2,4,6-trihydroxy acetophenone monohydrate, 2,5-dihydroxy acetophenone, α-cyano-4-hydroxycinnamic acid, picolinic acid, and 9-aminoacridine hemihydrate were purchased from (Sigma Aldrich, Gillingham, UK). Porcine Gb3 standard and *N*-heptadecanoyl ceramide trihexoside (C17:0) internal standard were purchased from Matreya (Pleasant Gap, PA, USA).

### Total Gb3 Standard

Total Gb3 (source: porcine RBC) was used for method development and standard curve purposes. Total Gb3 stock solution (200 ng/μL) was prepared by dissolving 10 mg of Gb3 standard in 50 mL of MeOH, then stored at –20 °C.

### Gb3 Internal Standard

*N*-heptadecanoyl ceramide trihexoside internal standard (0.5 mg) was dissolved in 1 mL of MeOH:CHCl_3_ (2:1) and then 9 mL of MeOH was added to generate 10 mL of internal standard at 50 ng/μL. This solution was stored at –20 °C in glass until used as 2 ng/μL.

### Extraction of Urinary Gb3

One hundred μL each of urine, MeOH containing 200 ng of Gb3 internal standard, and CHCl_3_ were mixed and incubated in a sonicating water bath for 5 min at room temperature. The mixture was then centrifuged at 13,000 rpm for 5 min and 1 μL of the lower (CHCl_3_) layer spotted directly onto a stainless steel 384-position MALDI target in triplicate.

### MALDI-TOF-MS

Once dry, the samples were overlaid with 1 μL of 5C2M matrix (saturated solution in 50%MeOH). MS and MS/MS (using laser induced dissociation in LIFT mode) were acquired on a Bruker Ultraflextreme TOF instrument equipped with a 1 kHz laser in positive ion mode. The MS spectra used for quantitation were the sum of 20,000 laser shots acquired in a random walk pattern. The peak areas used for Gb3 quantitation were extracted from the spectra using ClinproTools software (Bruker).

### Gb3 Internal Standard and Assay Calibration

*N*-heptadecanoyl ceramide trihexoside was used as the internal standard throughout this study. A standard curve was produced by extracting a series of Gb3 dilutions ranging from (0 to 40) ng/μL made in depleted urine from a healthy individual containing 2 ng/μL internal standard. The depleted urine was prepared by passing through a C18 cartridge and effective removal of Gb3 confirmed by parallel depletion of urine from a classic Fabry patient analyzed by MALDI-MS (Supplemental Information, Figure S-1). The four most abundant Gb3 species have been used to calculate the urinary total Gb3 level. The sodiated molecular ions of Gb3 species are at mass-to-charge ratios (*m/z*) of: 1130.7 (C22:0), 1146.7 (C22:0-OH), 1158.7 (C24:0), and 1174.7(C24:0-OH). The main peak of the Gb3 internal standard is at *m/z* 1060.7 (C17:0) with a secondary peak at *m/z* 1076.7 (C17:0-OH). The total area of both internal standard peaks and all four Gb3 species were used to determine the response ratio (Equation 1) and used in conjunction with a calibration curve to determine urinary Gb3 concentrations.

1 $$ Response\  ratio=\frac{total\  area\  of\  the\ 4\  most\  abundant\ Gb3\  species\ }{area\  of\ Gb3\  internal\  standard} $$

The limit of detection (LOD) and the limit of quantitation of total Gb3 in urine were determined using (Equation 2) and (Equation 3) respectively, where F = 3.3 and 10 for LOD and LOQ respectively, stdev = standard deviation and *b*: slope of the regression line (Supplemental Information Figure S-2).

2 $$ \mathrm{L}\mathrm{O}\mathrm{D}=\frac{\mathrm{F} \times \mathrm{stdev}}{\mathrm{b}} $$

3 $$ \mathrm{L}\mathrm{O}\mathrm{Q}=\frac{\mathrm{F} \times \mathrm{stdev}}{\mathrm{b}} $$

## Results and Discussion

### Optimization of Gb3 Extraction from Urine

**Figure 1:**
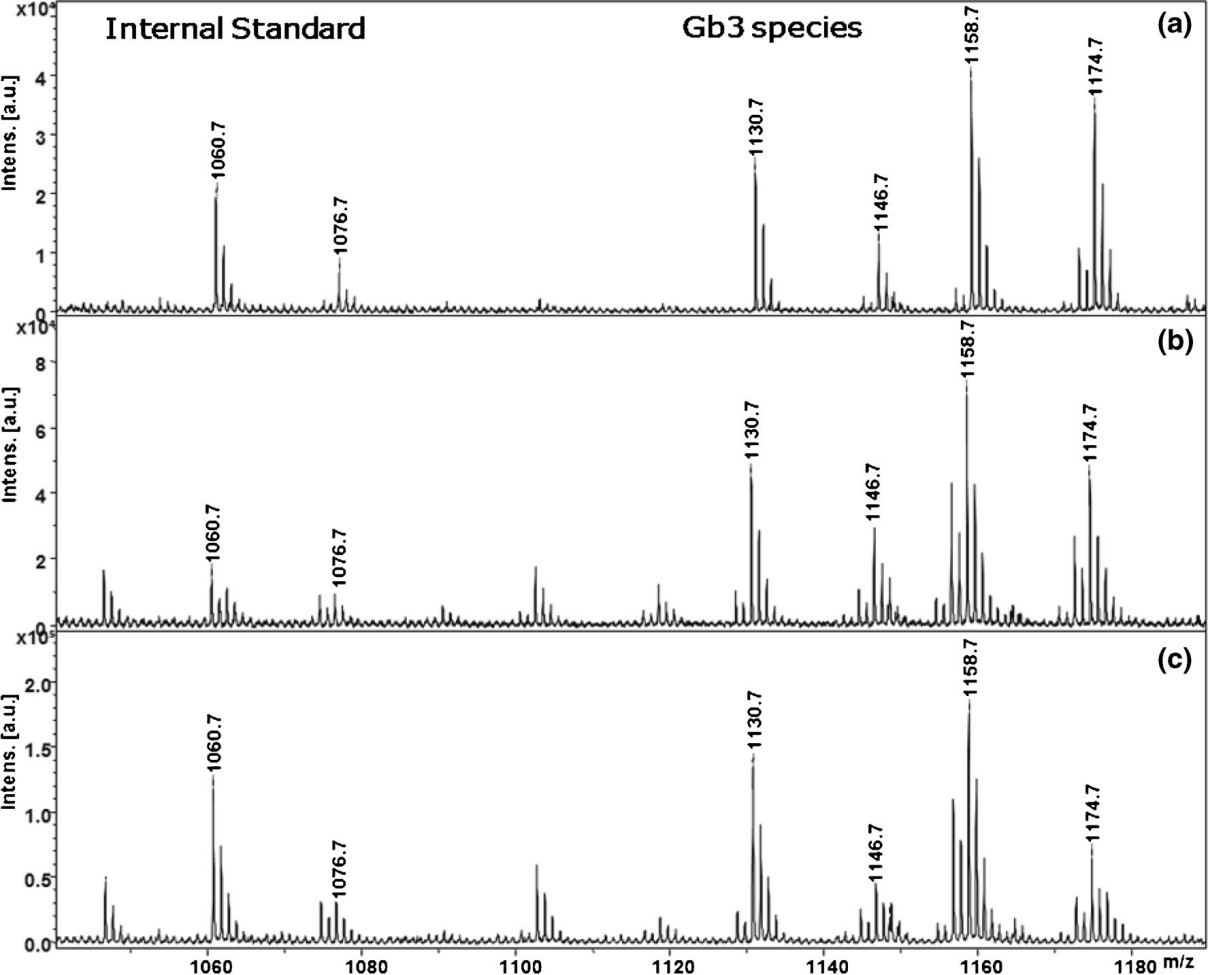
Validation of liquid-liquid extraction using different solvents: (**a**) acetone: MeOH:H_2_O, (45:45:10) (**b**) MeOH:CHCl_3_, (2:1) (**c**) MeOH:CHCl_3_ (1:1)

Depleted urine was spiked with 2.5 μL of porcine total Gb3 standard [200 ng/μL] generating a final concentration of 5 ng/μL, and the performance of various extraction procedures involving different volumes of MeOH, acetone, and CHCl_3_ were assessed by MALDI-TOF-MS. We found using 1:1:1 (v:v:v) of urine, MeOH, and CHCl_3_ to be the optimal condition for Gb3 extraction into the hydrophobic layer (CHCl_3_ layer) based on peak intensity and signal-to-noise ratio in the mass spectra (Figure 1) with no Gb3 detectable in the aqueous layer (Supplemental Information Figure S-3).

### Optimization of MALDI-TOF-MS

**Figure 2:**
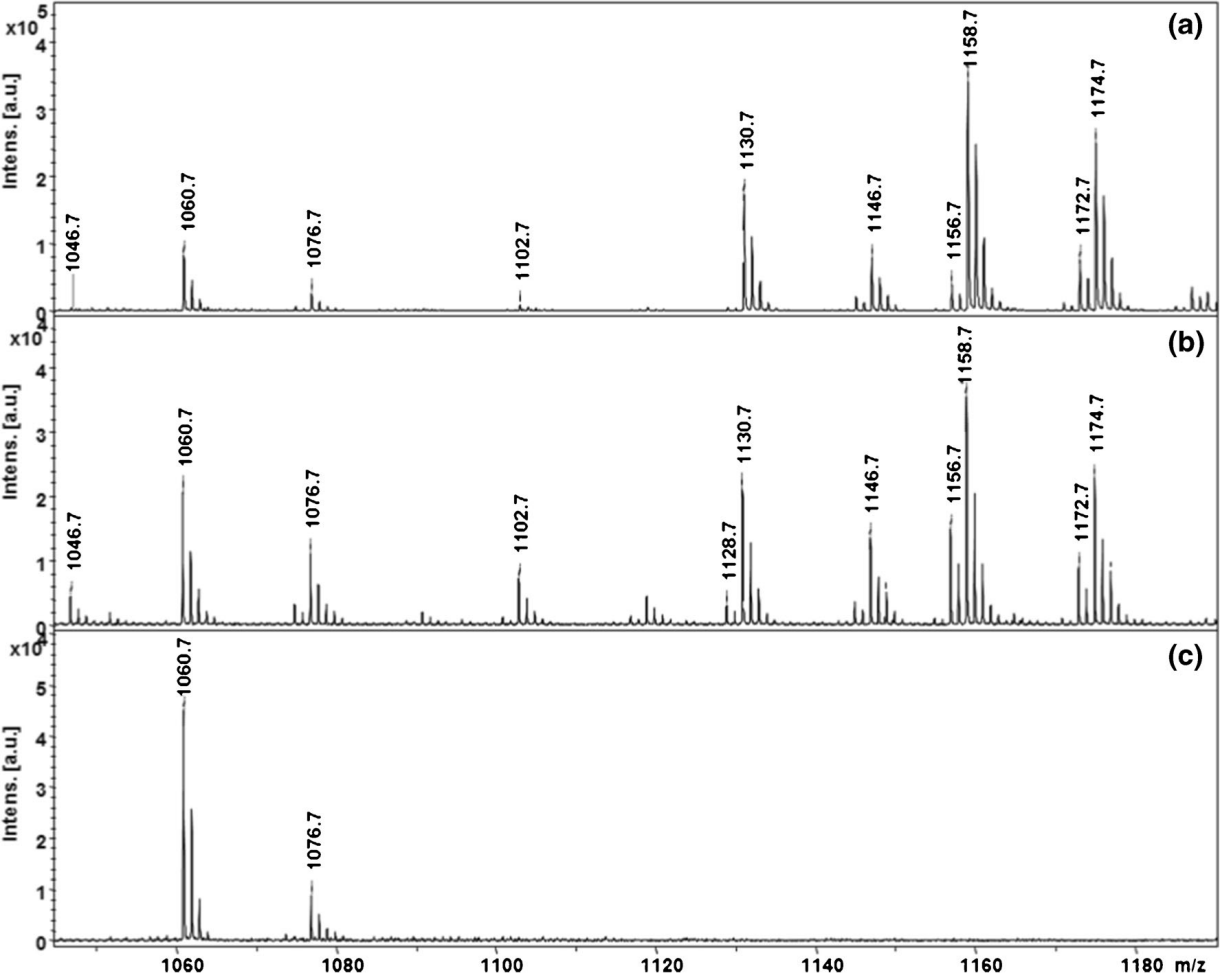
MS spectra of Gb3 species and Gb3 internal standard in (**a**) purchased porcine Gb3 standard, (**b**) urine from an untreated classic Fabry male, and (**c**) healthy control urine

We acquired and compared the MALDI spectra for depleted urine spiked with 5 ng/μL Gb3 using 12 different matrix compounds (spectra shown in Supplemental Information Figure S-4). The matrices were used as saturated solutions both in 50% MeOH and 50% ACN. Spectra obtained with 5C2M in 50% MeOH provided the best signal intensity and signal-to-noise ratio. Example MS spectra showing Gb3 peaks in the urine of a Fabry patient, porcine Gb3 standard, and a healthy control subject are shown in (Figure 2).

### MS/MS Confirmation of Gb3 Peaks in Fabry Patient Urine

**Figure 3:**
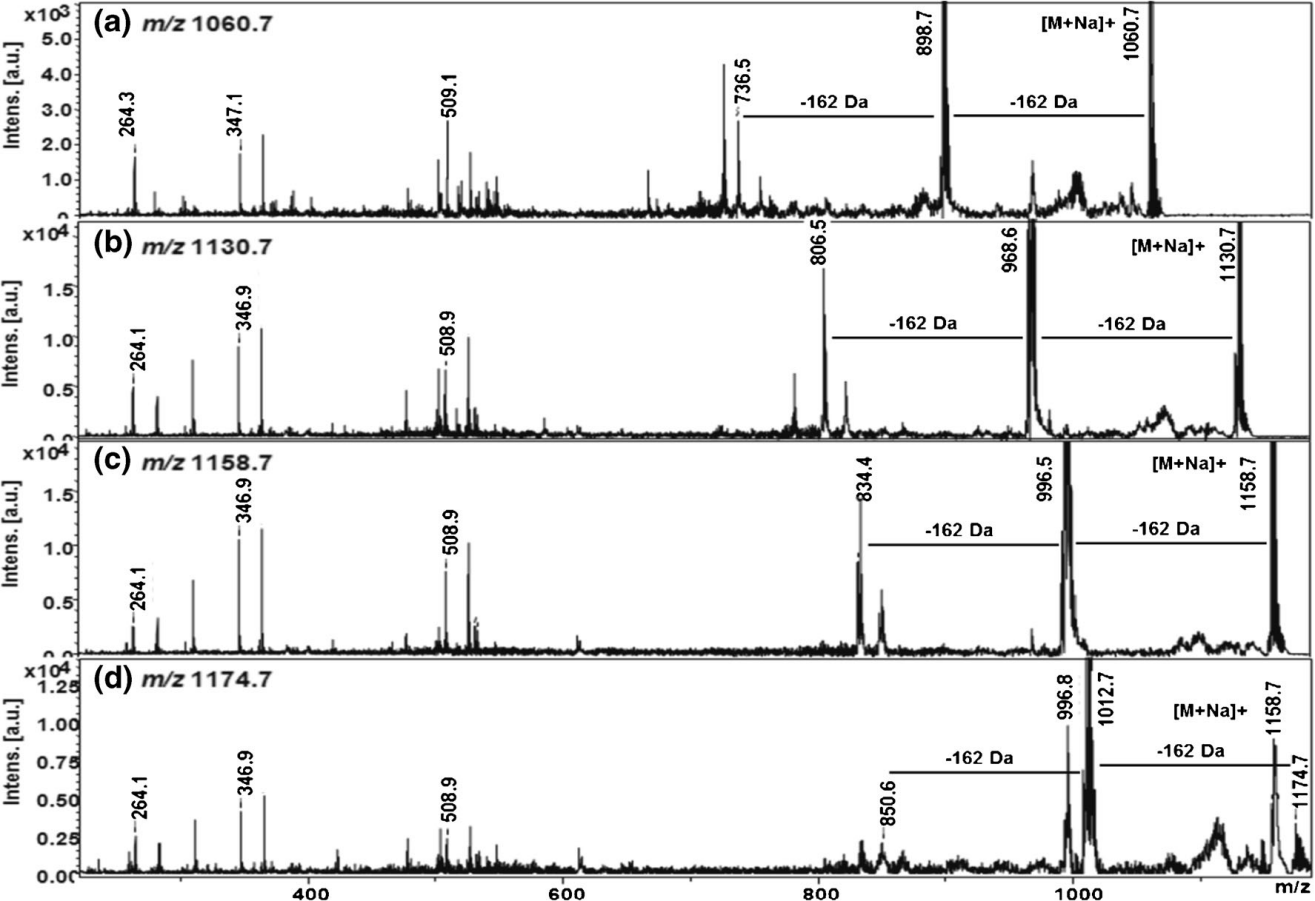
MALDI-TOF/TOF MS/MS of (**a**) Gb3-Internal standard *m/z* 1060.7 (C17:0); and the three most abundant Gb3 species in Fabry patient urine: *m/z* 1130.7 (C22:0), 1158.7 (C24:0), and 1174.7 (C24:0-OH). The peaks corresponding to ions generated by the loss of 1 or 2 sugars (–162 and –324 Da, respectively) have different *m/z* values for each species, whereas the fragments corresponding to sphingosine –2H_2_0, (*m/z* 264.1) and di- and tri-saccharide ions (*m/z* 347 and 509) are invariant

MS spectra of Fabry patient urine contain peaks with exactly the same *m/z* values as those in purchased porcine Gb3:*m/z* 1046.7 (predicted structure C16:0), 1074.7(C18:0), 1102.7(C20:0), 1128.7(C22:1), 1130.7(C22:0), 1146(C22:0-OH), 1156.7(C24:1), 1158.7(C24:0), 1172.7(C24:1-OH:), and 1174.7(C24:0-OH). To confirm that the peaks in Fabry patient urine spectra correspond to Gb3, they were subjected to MALDI-TOF/TOF MS/MS. The MS/MS spectra of the urine peaks show the loss of sugar moieties (Figure 3) and confirm that these ions correspond to Gb3 species by the presence of the fragment *m/z* 264.2, which represents sphingosine (sphingosine-2[H_2_O]). The purchased porcine Gb3 generated identical MS/MS spectra (data not shown).

### Quantitative Measurement of Urinary Gb3 by MALDI-TOF-MS

**Figure 4:**
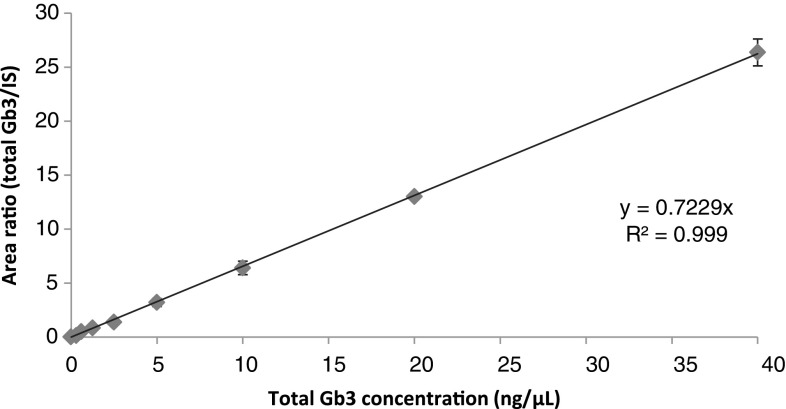
Gb3 standard curve. Increasing amounts of porcine Gb3 standard covering the reported clinical range and a constant amount of Gb3 internal standard were spiked into depleted urine from a healthy control subject. Thereafter, the lipids were extracted and analysed by MALDI-TOF MS

To correct for variations in extraction and ionization efficiencies an internal standard was added to all urine samples prior to extraction. The internal standard appears as a peak at *m/z* 1060.7 (C17:0) (plus a secondary peak at *m/z* 1076.7(C17:0-OH)). No peaks were seen at these *m/z* values in the MALDI spectra of any of the extracted urines used in this study in the absence of internal standard (Supplemental Information Figure S-5) (also, no contaminants with the same masses as the Gb3 peaks were detected). Gb3 concentrations were calculated from the (total Gb3/internal standard) area ratio as described in Methods and using the calibration curve shown in Figure 4. Inter-assay and intra-assay variability were measured to assess the reliability of the method. Urine samples of 10 healthy controls were used. Each sample was spiked with 2.5 μL of total Gb3 standard [200 ng/μL] generating a final concentration of 5 ng/μL. Each sample was processed as three independent technical replicates. Each extract was spotted onto the MALDI target in triplicate giving nine readings for each sample. The same procedure was repeated on three different days resulting in each sample being analyzed 27 times. The results showed high reproducibility and reliability of the method with an intra-assay coefficient of variation of 9.9% and inter-assay of 13.7%. The lower limit of detection was 0.15 ng/μL, and limit of quantitation was 0.30 ng/μL. The assay was linear up to the highest Gb3 concentration investigated (40 ng/μL).

### Urinary Gb3 Levels in Fabry Patients

**Figure 5:**
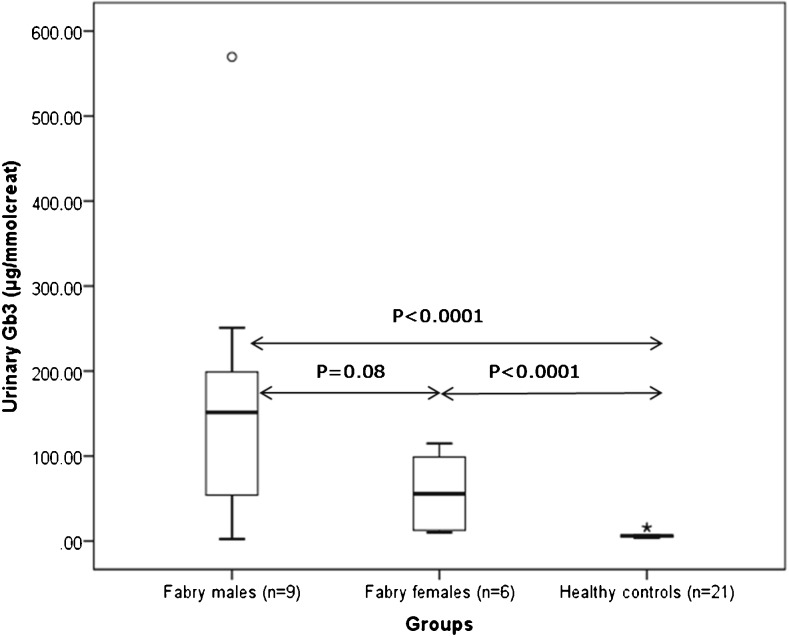
Urinary total Gb3 levels: box-and-whiskers plot showing urinary Gb3 levels in both genders of classical Fabry patients and healthy control subjects (*P* values obtained using *t*-tests)

We measured the levels of urinary Gb3 in classical Fabry patients (n = 15) and healthy controls (n = 21) as shown in Figure 5. The mean Gb3 concentrations in classic patients and healthy controls were 1.35 ng/μL and 0.08 ng/μL, respectively. Prior to statistical comparison, the urinary Gb3 concentrations were normalized to urinary creatinine. Overall, the mean normalized concentration in the classic Fabry patients was significantly higher than healthy controls (n = 21, mean = 6.7 $\upmu \mathrm{g}/\mathrm{mmol}\;\mathrm{creat}$), (*p* < 0.0001). The normalized mean concentration in the classic Fabry males was higher than in Fabry females (171.7 v 57.9 $\upmu \mathrm{g}/\mathrm{mmol}\;\mathrm{creatinine}\Big)\kern0.1em \mathrm{although}\;\mathrm{this}\;\mathrm{did}\;\mathrm{not}\;\mathrm{reach}\;\mathrm{statistical}\;\mathrm{significance}$ (*p* = 0.08), and urinary Gb3 was significantly higher in both male and female FD patients than in healthy controls (*p* < 0.0001 in both cases). Using the urinary Gb3 concentrations measured in the 36 individuals in this study to detect Fabry disease generated a receiver operator characteristic (ROC) curve with an area of 0.92 (95% CI 0.79–1.00) and, using a threshold of 25 μg/mmol creatinine gave a sensitivity of 80% (95% CI 52–96) at 100% specificity (95% CI 84–100), (Supplemental Information Figure S-6).

Gb3 generates multiple peaks in mass spectra. The total area of the four most intense peaks [*m/z* 1130.7 (C22:0), 1146.7 (C22:0-OH), 1158.7 (C24:0), and 1174.7(C24:0-OH)] was used to measure the ratio of Gb3 to the internal standard. These four peaks were selected because they have the highest intensities, and they were detected in all patients whereas the other peaks (other Gb3 species) were not always detected. We found that the ratio of these four peaks to one another is constant across individuals (i.e. classic FD results in accumulation of all 4 Gb3 species). Although we have used the sum of the four main peaks for quantitation (considering that this might be the most robust approach), we also found that any one of the four peaks alone would yield the same results (data not shown).

## Discussion

By coupling a novel liquid–liquid extraction and MALDI-TOF-MS with an internal standard, we have been able to develop a rapid (<15 min from start to finish) method for measuring Gb3 in urine. Considerable optimization of the Gb3 extraction and MS analysis has been performed to produce an assay with good reproducibility, sensitivity, and specificity sufficient to easily detect the elevated levels of urinary Gb3 that are expected in classic Fabry patients. *N*-heptadecanoyl ceramide trihexoside has been used as an internal standard in this study because it has very similar chemical properties to human Gb3 but cannot be synthesized by humans (due to the odd number of carbon atoms). The internal standard generates a main peak at *m/z* 1060.7 (C17:0) and a minor peak at *m/z* 1076.7 (C17:0-OH) (most likely due to oxidation, both peaks were combined in calculations). Following liquid–liquid extraction human urine is devoid of peaks at these *m/z* values unless the internal standard is added (Supplemental Information Figure S-5). Including the internal standard in our assay has enabled a high degree of reducibility and reliability to be reached with correspondingly low intra-assay and inter-assay coefficients of variation. The assay is based on MS; however, MS/MS was used to verify the identity of the Gb3 peaks: the most abundant peaks at *m/z* 1130.7 (C22:0), 1146.7 (C22:0-OH), 1158.7 (C24:0), and 1174.7 (C24:0-OH) all show identical fragmentation pattern by losing sugar groups (–162 Da) and generating the di-dehydrated sphingosine moiety (*m/z* 264.2) as previously reported for Gb3 [13, 14, 16, 18, 22].

## Conclusion

Our method significantly reduces the number of steps and time required to complete the test compared with the current standard methods. The method could be easily implemented in any laboratory with access to a MALDI mass spectrometer and used for noninvasive, cost-effective detection of Fabry disease.

## Electronic supplementary material

Table S1 (DOCX 18 kb)

Table S2 (DOCX 19 kb)

Figure S-1 (DOCX 103 kb)

Figure S-2 (DOCX 63 kb)

Figure S-3 (DOCX 57 kb)

Figure S-4 (DOCX 350 kb)

Figure S-5 (DOCX 60 kb)

Figure S-6 (DOCX 28 kb)
