# Supplementary material for: A Novel Rapid MALDI-TOF-MS-Based Method for Measuring Urinary Globotriaosylceramide in Fabry Patients
Source: J Am Soc Mass Spectrom. 2016 Jan 21;27:719–25. doi: 10.1007/s13361-015-1318-4 (PMC4792351; doi:10.1007/s13361-015-1318-4)
Supplement: Supplementary file 1 — (DOCX 18 kb) [file 13361_2015_1318_MOESM1_ESM.docx]

|  | **Fabry patients** | | | | **Healthy controls** | |
| --- | --- | --- | --- | --- | --- | --- |
|  | **Hemizygous males** | | **Heterozygous females** | | **Male** | **Female** |
|  | **Untreated** | **Treated** | **Untreated** | **Treated** |  |  |
| **Numbers** | 2 | 7 | 4 | 2 | 17 | 4 |
| **Age (mean, y)** | 36.5±26.2 | 41.4±12.0 | 70.3±11.4 | 66.0±5.7 | 50.1±20.3 | 67.8±13. 9 |
| **Age (range, y)** | 18-55 | 19-52 | 56-80 | 62-70 | 20-82 | 51-85 |

**Table-S1: Anthropometric data of Fabry patients and healthy controls**.
