# Supplementary material for: A Novel Rapid MALDI-TOF-MS-Based Method for Measuring Urinary Globotriaosylceramide in Fabry Patients
Source: J Am Soc Mass Spectrom. 2016 Jan 21;27:719–25. doi: 10.1007/s13361-015-1318-4 (PMC4792351; doi:10.1007/s13361-015-1318-4)
Supplement: Supplementary file 2 — (DOCX 19 kb) [file 13361_2015_1318_MOESM2_ESM.docx]

| **Analyte**  **structure** | **Gb3 species *m/z***  **(protonated ion)** | **Gb3 species detected *m/z***  **(monosodiated ion)** | **Predicted formula**  **(monosodiated ion)** |
| --- | --- | --- | --- |
| C16:0 | 1024.7 | 1046.7 | C52H97NO18Na |
| C17:0 (IS) | 1038.7 | 1060.7 | C53H99NO18Na |
| C18:0 | 1052.7 | 1074.7 | C54H101NO18Na |
| C20:0 | 1080.7 | 1102.7 | C56H105NO18Na |
| C22:1 | 1106.7 | 1128.7 | C58H107NO18Na |
| C22:0 | 1108.7 | 1130.7 | C58H109NO18Na |
| C22:0-OH | 1124.7 | 1146.7 | C58H109NO19Na |
| C24:1 | 1134.7 | 1156.7 | C60H111NO18Na |
| C24:0 | 1136.7 | 1158.7 | C60H113NO18Na |
| C24:1-OH | 1150.7 | 1172.7 | C60H111NO19Na |
| C24:0-OH | 1152.7 | 1174.7 | C60H113NO19Na |

**Table-S2: Gb3 ions:** The table shows the calculated m/z values for the singly charged protonated parent ions of Gb3 species, the measured m/z values (monosodiated ions) and the predicted chemical formulae.
