# Supplementary material for: A Novel Rapid MALDI-TOF-MS-Based Method for Measuring Urinary Globotriaosylceramide in Fabry Patients
Source: J Am Soc Mass Spectrom. 2016 Jan 21;27:719–25. doi: 10.1007/s13361-015-1318-4 (PMC4792351; doi:10.1007/s13361-015-1318-4)
Supplement: Supplementary file 3 — (DOCX 103 kb) [file 13361_2015_1318_MOESM3_ESM.docx]

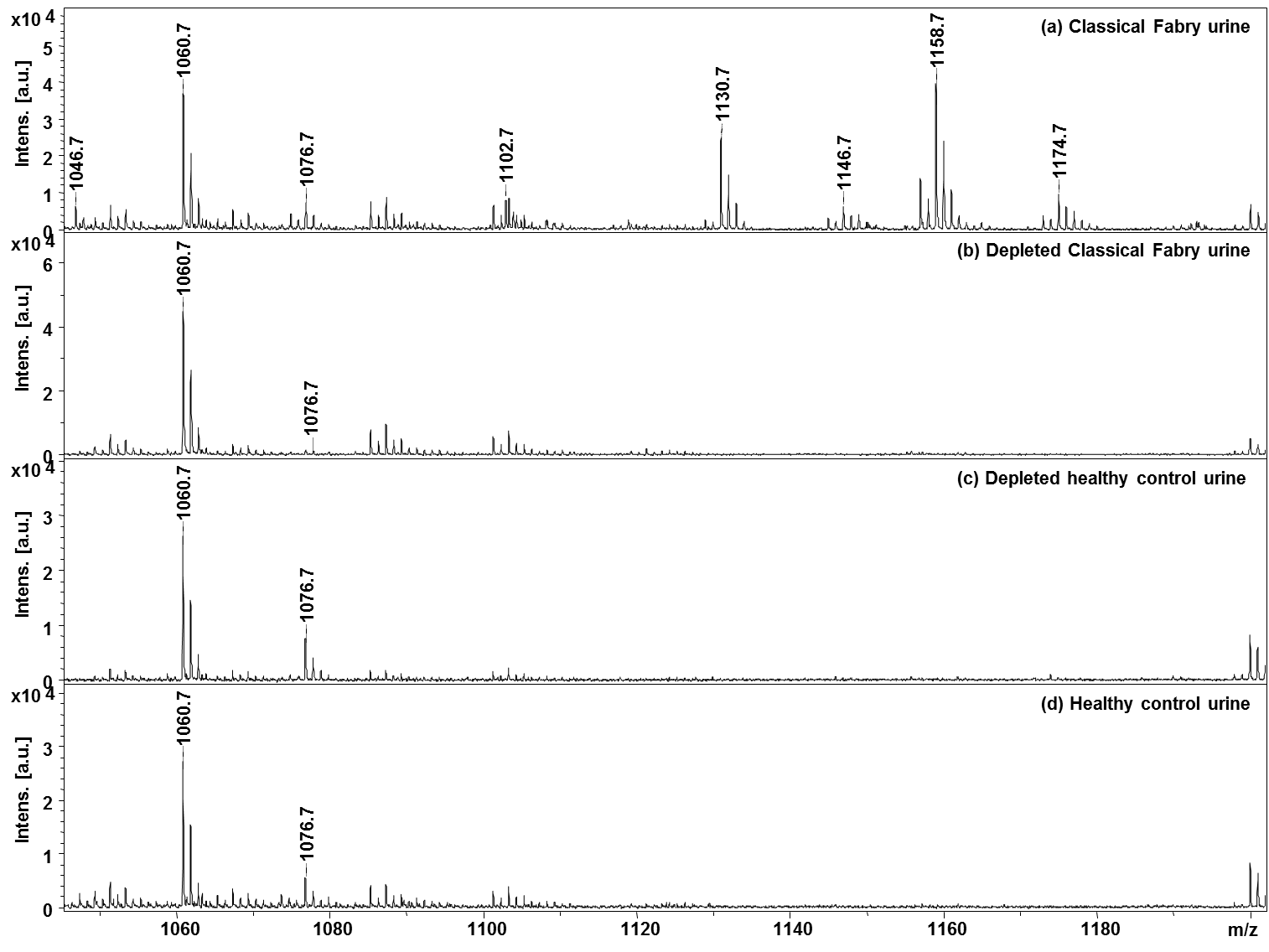


**Figure S-1: Validation of Gb3 depletion:** MS spectra of urine samples pre- and post-depletion. (a) Urine from classical Fabry patient pre-depletion. (b) Depleted urine from classical Fabry patient post-depletion. (c) Urine from a healthy control post-depletion. (d) Urine from healthy control pre-depletion. Gb3 internal standard is present in all spectra because it has been added post-depletion.
