# Supplementary material for: A Novel Rapid MALDI-TOF-MS-Based Method for Measuring Urinary Globotriaosylceramide in Fabry Patients
Source: J Am Soc Mass Spectrom. 2016 Jan 21;27:719–25. doi: 10.1007/s13361-015-1318-4 (PMC4792351; doi:10.1007/s13361-015-1318-4)
Supplement: Supplementary file 4 — (DOCX 63 kb) [file 13361_2015_1318_MOESM4_ESM.docx]

**
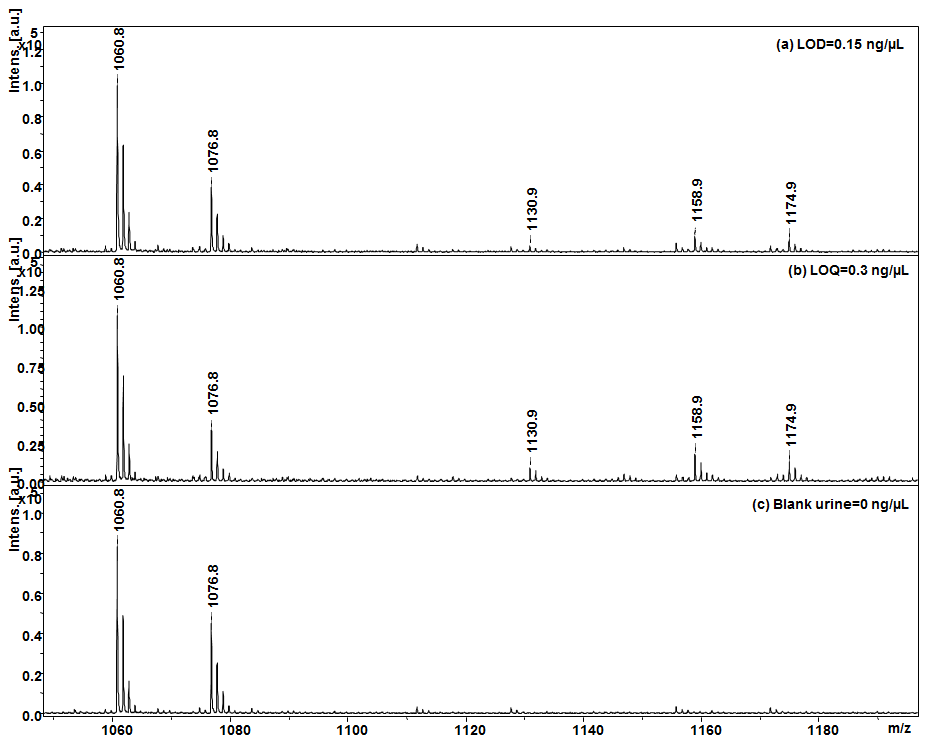
**

**Figure S-2: LOD and LOQ of urinary total Gb3: (a)** MS spectra of urinary Gb3 at the LOD. **(b)** MS spectra of urinary Gb3 at the LOQ. **(c)** MS spectra of depleted urine.
