# Supplementary material for: A Novel Rapid MALDI-TOF-MS-Based Method for Measuring Urinary Globotriaosylceramide in Fabry Patients
Source: J Am Soc Mass Spectrom. 2016 Jan 21;27:719–25. doi: 10.1007/s13361-015-1318-4 (PMC4792351; doi:10.1007/s13361-015-1318-4)
Supplement: Supplementary file 5 — (DOCX 57 kb) [file 13361_2015_1318_MOESM5_ESM.docx]

**
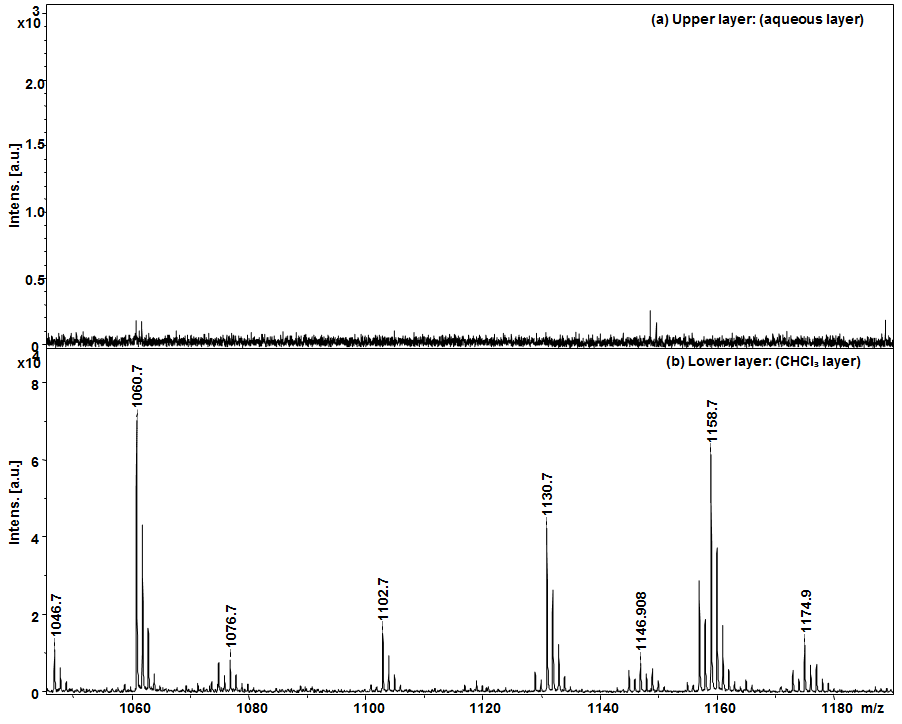
**

**Figure S-3: Validation of liquid-liquid extraction.** MALDI-TOF**-**MS spectra of Gb3 species and Gb3 internal standard in: **(a)** the aqueous layer (upper layer) and **(b)** Chloroform layer (lower layer). Gb3 species and Gb3 internal standard were spiked in normal urine from a healthy control subject.
