# Supplementary material for: A Novel Rapid MALDI-TOF-MS-Based Method for Measuring Urinary Globotriaosylceramide in Fabry Patients
Source: J Am Soc Mass Spectrom. 2016 Jan 21;27:719–25. doi: 10.1007/s13361-015-1318-4 (PMC4792351; doi:10.1007/s13361-015-1318-4)
Supplement: Supplementary file 6 — (DOCX 350 kb) [file 13361_2015_1318_MOESM6_ESM.docx]

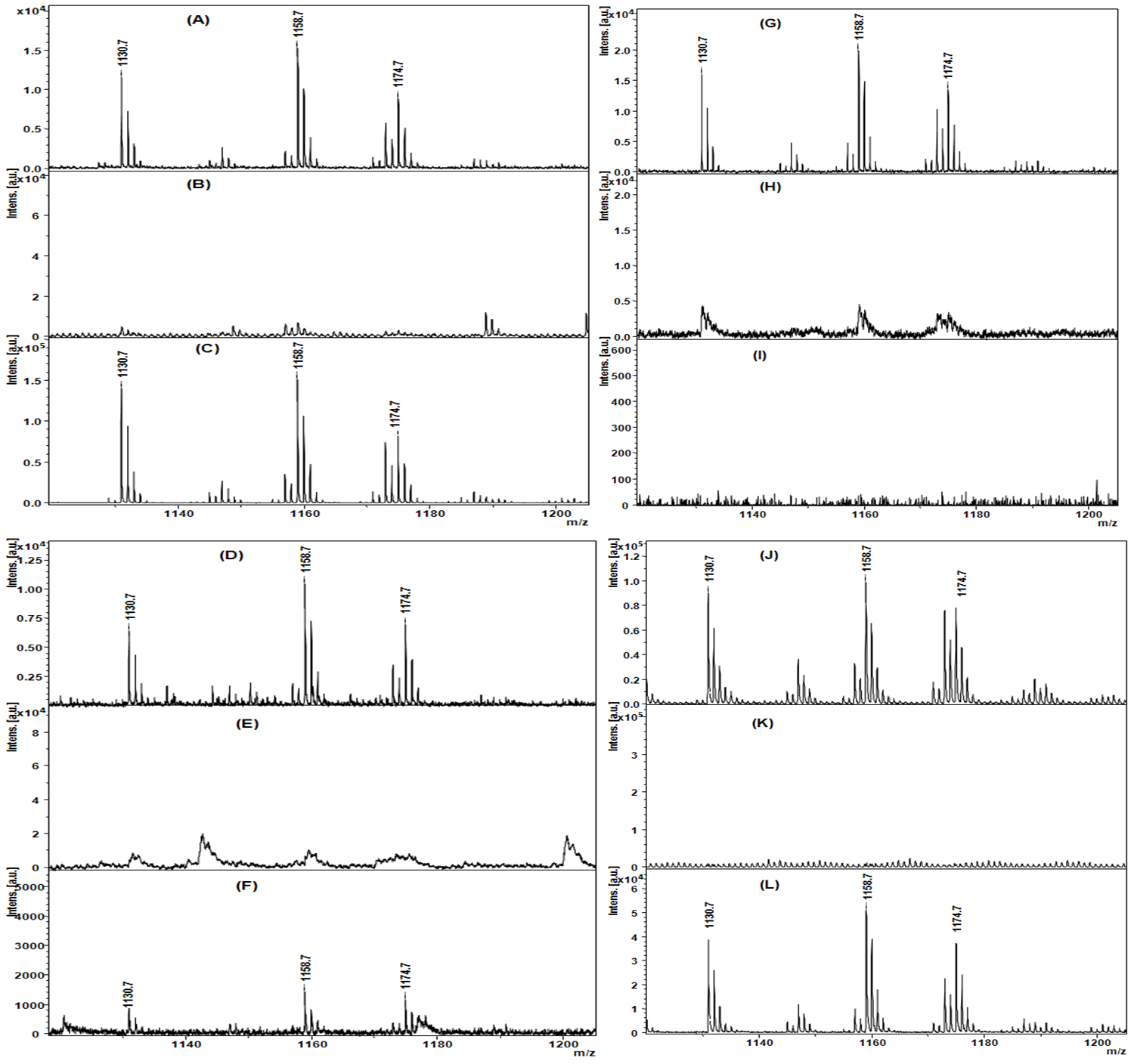


**Figure S-4: Evaluation of different matrix compounds**. MALDI-TOF spectra of porcine Gb3 generated using 12 different matrix compounds prepared as saturated solutions in 50%MeOH are shown. The *m/z* range covers the mass range of the most intense 3 peaks of Gb3 (*m/z* 1158.7, 1174.7 & 1130.7). These matrix compounds used were: (A) 2-(4-hydroxyphenylazo) benzoic acid, (B) 2-mercaptobenzothiazole, (C) 5-chloro-2-meracptobenzothiazole, (D) 2,5-dihydroxy acetophenone, (E) 6aza-2-thiothymine, (F) 9-aminoacridine hemihydrate, (G) α-cyano-4-hydroxycinnamic acid, (H) 2.5-Dihysroxybenzoic acid (DHB), (I) picolinic acid, (J) super-DHB, (K) sinapic acid, (L) 2,4,6-trihydroxy acetophenone monohydrate.
