# Supplementary material for: A Novel Rapid MALDI-TOF-MS-Based Method for Measuring Urinary Globotriaosylceramide in Fabry Patients
Source: J Am Soc Mass Spectrom. 2016 Jan 21;27:719–25. doi: 10.1007/s13361-015-1318-4 (PMC4792351; doi:10.1007/s13361-015-1318-4)
Supplement: Supplementary file 7 — (DOCX 60 kb) [file 13361_2015_1318_MOESM7_ESM.docx]

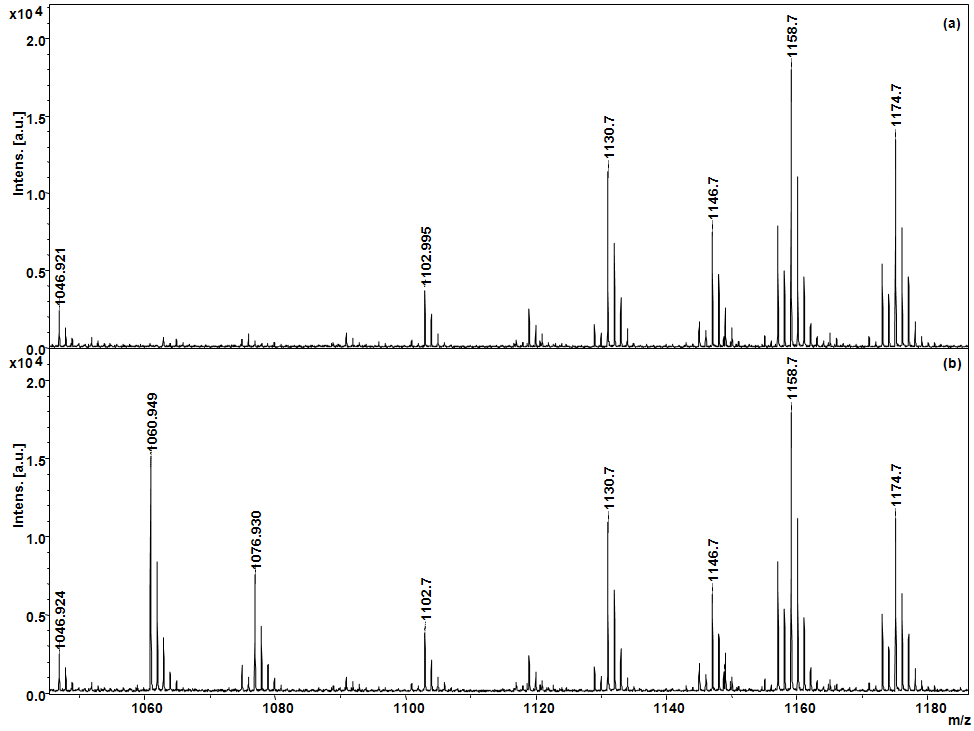


**Figure S-5: Gb3 internal standard in human urine:** (a) Fabry urine without adding internal standard. (b) Fabry urine spiked with Gb3 internal standard.
