# Supplementary figures and images for: A Novel Rapid MALDI-TOF-MS-Based Method for Measuring Urinary Globotriaosylceramide in Fabry Patients
Source: J Am Soc Mass Spectrom. 2016 Jan 21;27:719–25. doi: 10.1007/s13361-015-1318-4 (PMC4792351; doi:10.1007/s13361-015-1318-4)

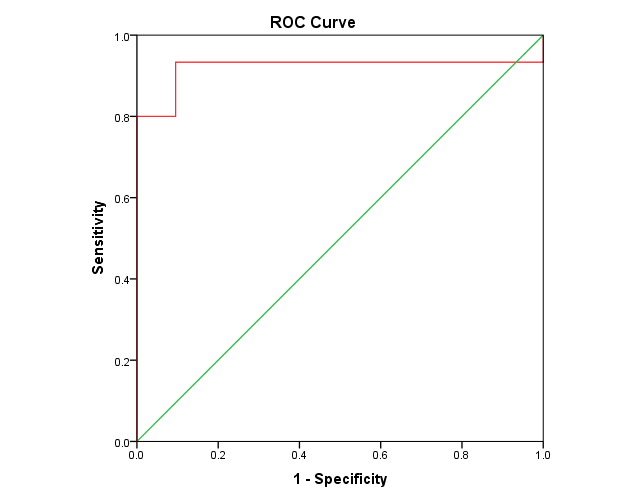


**Figure S-6:** ROC curve of Urinary Gb3 in Fabry patients vs. healthy controls

Supplement: Supplementary file 8 — (DOCX 28 kb) [file 13361_2015_1318_MOESM8_ESM.docx]
